# Parenting and Occupational Therapy: An Exploration of Global Practice

**DOI:** 10.1177/15394492251382465

**Published:** 2025-11-14

**Authors:** Margaret McGrath, Anne Honey, Fidaa Almomani, Yu-Wei Ryan Chen, Yvonne Codd, Junghun Aj Kim, Masafumi Kunishige, Rodolfo Morrison, Jessica Peterson, Evelina Pituch, Muhammad Hibatullah Romli, Deena Rozen, Rachel Sabbah, Hassan I. Sarsak, Elaine Saunders, So Sin Sim, Hwei Lan Tan, Farahiyah Wan Yunus, Wong Wing Tung, Veronica O. Mara, John V. Rider

**Affiliations:** 1 University College Cork, Ireland; 2 The University of Sydney, Camperdown, New South Wales, Australia; 3 Jordan University of Science and Technology, Ar-Ramtha, Jordan; 4 Trinity College Dublin, Ireland; 5 Kangwon National University, Gangwon-do, South Korea; 6 Bunkyo Gakuin University, Saitama, Japan; 7 Universidad de Chile, Santiago, Chile; 8 Matrescence Occupational Therapy, Austin, TX, USA; 9 University of Toronto Scarborough, ON, Canada; 10 Universiti Putra Malaysia, Serdang, Selangor, Malaysia; 11 Meuhedet Health Services, Israel; 12 Batterjee Medical College, Jeddah, Saudi Arabia; 13 Brunel University of London, UK; 14 Singapore Institute of Technology, Singapore; 15 Universiti Kebangsaan Malaysia, Kuala Lumpur, Malaysia; 16 Touro University Nevada, Henderson, NV, USA

**Keywords:** occupational therapy, services, survey, parenting, family-centered practice

## Abstract

Occupational therapists recognize parenting as within their scope; however, little is known about how this translates to practice with adult clients with disabilities or other challenges. We sought to describe contemporary global occupational therapy practice for parenting with adult clients, including assessment and interventions. A cross-sectional e-survey designed for the study was implemented in eight languages. Participants were recruited using convenience sampling. Responses were received from 1,357 occupational therapists across 42 countries. Of these, 43.1% (*n* = 586) frequently ask their clients who are parents about parenting roles and occupations, while 34.2% (*n* = 465) frequently or very frequently address parenting concerns. Assessments typically relied on informal approaches or the use of broad measures of occupational performance. The most frequently reported parenting intervention was the provision of education and training in parenting skills. Occupational therapy practice for parenting remains underdeveloped, with limited evidence of comprehensive occupational therapy assessment or intervention.

## Plain Language Summary

How do occupational therapists support clients who experience challenges with parenting roles and occupations: Results from a global e-survey

Adults living with illness or disabilities often describe difficulties and unmet needs in performing parenting roles. Occupational therapists could potentially address these concerns, but little is known about how they do so when working with adults. We conducted an online survey of occupational therapists working with adults to explore if and how they address parenting in their everyday work. The survey was designed for the study and is available online in eight languages. It included questions about how often therapists asked about parenting, their assessment and intervention practices, and their prior training in occupational therapy for parents. We invited occupational therapists who worked with adults (18 years and older) to participate. A total of 1,357 therapists from 42 countries completed the survey. Just over one third of respondents frequently or very frequently asked clients about parenting concerns or provided occupational therapy to address them. Prior training in occupational therapy and parenting increased the likelihood of asking about and addressing parenting concerns. The most common interventions involved education and training in parenting skills. However, few therapists reported using comprehensive assessment or intervention approaches for clients experiencing parenting challenges. Overall, the results suggest that occupational therapy practice related to parenting is underdeveloped. To better support adults facing barriers to parenting, there is a clear need to develop and implement targeted occupational therapy assessments and interventions focused on parenting roles.

## Introduction

Parenting is a highly complex and culturally situated endeavor, and many parents report challenges in performing parenting roles and occupations in line with their own or society’s expectations (Battalova, 2019). Occupational therapists recognize the value and complexity of parenting roles and occupations (Lim et al., 2022; McGrath et al., 2024; Rider & Selim, 2022). Lim et al.’s (2022) Parenting Occupations and Purposes Framework identifies more than 100 specific parenting occupations, ranging from basic child care tasks such as bathing, diapering, and feeding, to more complex tasks such as managing the child’s social networks through facilitating relationships with family members and organizing playdates with peers. The individual and societal value of parenting is acknowledged in occupational therapy literature (Lampe et al., 2019). Parenting shapes children’s social, emotional, intellectual, and behavioral development and influences society through forming the next generation (McGrath et al., 2024). Furthermore, addressing parenting has been identified as within the scope of occupational therapy practice (American Occupational Therapy Association, 2020). Despite this recognition, traditional occupational therapy practice with parents has been largely restricted to collaboration with parents in the context of providing services for children—a child-centric approach. Far less attention has been paid to supporting adults with disabilities and other challenges to engage in the parenting occupations they need and want to do—a parent-centric approach (McGrath et al., 2024).

This is surprising given that many parents living with disabilities report unmet parenting support needs (Codd et al., 2023; Pituch et al., 2022; Rider & Selim, 2022) and would welcome input from health professionals in managing parenting roles (Honey et al., 2024; Codd et al., 2023; Pituch et al., 2024). These needs may arise from a range of factors, including difficulties in performing parenting tasks due to physical or cognitive impairments (Sparud-Lundin & Berg, 2011; Williams et al., 2019), challenges with emotional regulation (Chen et al., 2021; Griffiths et al., 2019), difficulties managing boundaries, establishing routines, or coping with fatigue (Dahlberg & Berg, 2019; Stransky et al., 2024), and side effects associated with medication use (Ackerman et al., 2015; Williams et al., 2019). Additional concerns may include worries about the impact of a parent’s disability on their child (Hailey et al., 2019; Pihkala & Johansson, 2008; Schiena et al., 2019; Semple & McCance, 2010), as well as reduced parental self-efficacy resulting from negative societal attitudes toward parents with disabilities, insufficient social support, and fears related to custody loss (Bartsch et al., 2016; Cammidge et al., 2016; Sparud-Lundin & Berg, 2011).

Occupational therapists can use their expertise in occupational analysis to support parents in adapting parenting tasks or modifying the environment in which parenting occurs to accommodate impairments and facilitate successful performance of parenting roles. Occupational therapists can also support parents in balancing the competing demands of parenthood with the management of long-term health conditions, helping parents to minimize the impact of their health condition on children. However, the profile of occupational therapy practice as it relates to parents remains elusive and poorly understood (Wint et al., 2016). Lampe et al. (2019) explored occupational therapy practice with parents with physical disability in the United States and identified a number of gaps in approaches to assessment and intervention. While to our knowledge this is the only published survey of occupational therapy practice and parenting, the extent to which Lampe et al.’s (2019) findings can be generalized remains unclear, given its scope and the relatively small participation rate (*n* = 51).

Given the number of parents living with disabilities, there is a clear need to better understand current occupational therapy practices supporting this population (McGrath et al., 2024). This study aimed to gather a broad range of perspectives from occupational therapy practitioners regarding their practice relating to parenting roles and occupations. Specifically, the study explored (a) the extent to which occupational therapists working with adults address parenting within their practice, (b) the methods used by therapists to assess parenting roles and related occupations, and (c) the types of interventions offered to support parents in managing challenges associated with their parenting responsibilities.

## Method

### Study Design

A cross-sectional study using Research Electronic Data Capture (REDCap) online survey (Harris et al., 2019; Harris et al., 2009) hosted at the University of Sydney was used to collect global data from a convenience sample of occupational therapists. Study design and reporting were guided by the Checklist for Reporting Results of Internet E-Surveys (CHERRIES) (Eysenbach, 2004). Occupational therapists were eligible to participate if they were working as an occupational therapist with adults (those aged 16 years and above) as a primary client group in the past 5 years. No limitations were placed on the type or location of practice or the type of disability.

Potential participants were given a survey link configured to enable completion with a computer or mobile device. Participants could choose to complete the survey in one of nine languages. The survey included a combination of fixed-choice and free-text answers and took approximately 15 minutes to complete.

#### Survey Development

**Figure 1.**
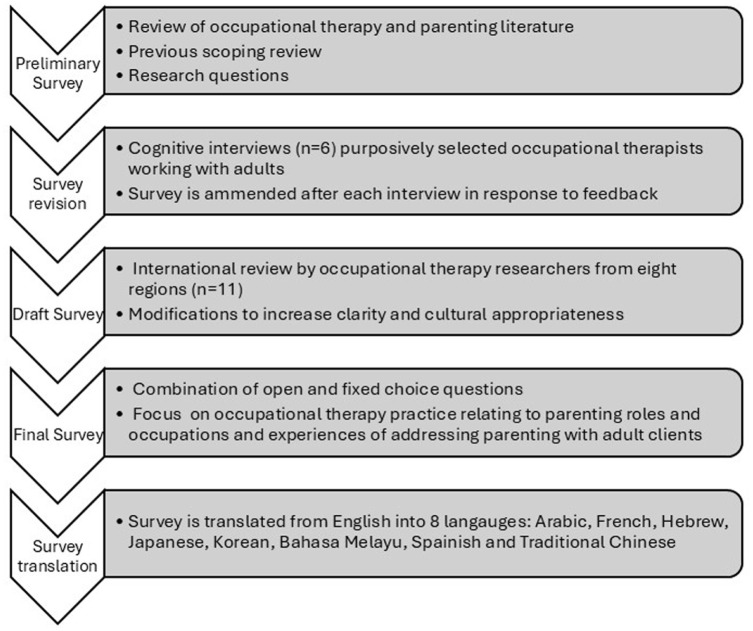
Survey Development and Translation Process.

As no existing instruments examined occupational therapy practice in parenting, a survey was developed specifically for this study (see Figure 1). The preliminary survey was informed by existing literature, research questions, and a prior scoping review. Refinement was undertaken through cognitive interviews (Acquadro et al., 2008; Beatty & Willis, 2007; Collins, 2015) with six occupational therapists from diverse practice backgrounds and experience levels (ranging from 2 to 25+ years). Participants completed the draft survey online while verbalizing their thoughts aloud, allowing researchers to assess clarity, relevance, and usability. Interviews were recorded (with consent), and detailed field notes were taken. Revisions included rewording, combining items, clarifying terms, modifying response options, and adding instructions. Subsequently, international OT academics from 11 countries reviewed the survey for clarity and cultural relevance, prompting further modifications. The final version included open- and fixed-choice questions and used branching logic. It was translated into eight languages using forward and backward translation by bilingual occupational therapy professionals, with discrepancies resolved by consensus.

### Recruitment and Data Collection

Our research team included members from 14 different countries. Drawing on our team’s global reach, relevant organizations and leaders in occupational therapy internationally were emailed the survey with a request to distribute it among their networks. Participants were also recruited via social media platforms, including X, Facebook, Instagram, and LinkedIn. Respondents were encouraged to share the survey through their professional networks (e.g., emails, e-newsletters, and web postings). The survey was open from April to December 2023.

#### Response Rate

A total of 1,734 participants commenced the study. Of these, 45 did not complete the online information and consent form, and 275 did not meet one or more of the inclusion criteria. A further 57 responses were excluded as they only completed a small number of questions, or the responses were considered unreliable based on repeated or apparent duplicate responses. This resulted in 1,357 participants with complete and authentic data whose responses were included in the final analysis.

### Data Analysis

Data were downloaded from REDCap and analyzed using SPSS (Version 29.01.1). All data were screened, and no outliers were identified. An overview of the sample’s assessment and intervention practices in relation to parenting was generated using descriptive statistics. Multinominal logistic regression was used to determine if the likelihood of participants (a) asking clients who were parents about parenting roles and occupations or (b) assessing parenting roles and occupations could be predicted by variation in region of practice, years of experience as an occupational therapist, additional training in occupational therapy for parenting roles and occupations or percentage of adult clients known to be parents. Prior to conducting the multinomial logistic regression, data were checked to ensure that the assumptions of independence, collinearity, and absence of outliers were met.

### Ethics

Ethical approval for the study was granted by the Human Research Ethics Committee at the University of Sydney (2022/898). Participation was voluntary, anonymous, and no incentives were offered. All participants were provided with a participant information statement on opening the survey link. Consent to participate in the study was implied by submission of the survey.

## Results

### Overview of Participants and Practice Context

Participants’ demographic and practice-related characteristics are presented in Table 1. We recruited participants practicing in 42 different countries (see online Appendix 1 for breakdown). To support further analysis, we categorized individual countries according to the eight regions recognized by the World Bank (The World Bank, n.d.). Most responses were received from therapists working in the Middle East and North Africa (*n* = 581, 42.8%), followed by East Asia and Pacific (*n* = 284, 20.9%) and North America (*n* = 203, 15.0%). A small number of responses were received from therapists practicing in sub-Saharan Africa (*n* = 5, 0.4%) and South Asia (*n* = 3, 0.2%). Given the small number of respondents from these regions, they were excluded from further analysis.

**Table 1.** Summary of Participants’ Demographic and Practice-Related Characteristics (N = 1,357).

Variable		*n*	%
Gender			
	Female	1,045	77.0
	Male	291	21.4
	Non-binary	6	0.4
	Transgender	4	0.3
	Prefer not to respond	8	0.6
	Missing	3	0.2
Region			
	East Asia and Pacific	285	21
	Europe and Central Asia	172	12.7
	Latin America and the Caribbean	99	7.3
	Middle East and North Africa	581	42.8
	North America	203	15.0
	South Asia	3	0.2
	Sub-Saharan Africa	5	0.4
	Missing	9	0.7
Years working as an occupational therapist			
	0–2 years	370	27.3
	2–5 years	258	19.0
	6–10 years	259	19.1
	11–20 years	256	18.9
	>20 years	209	15.4
	Missing	5	0.4
Received specific training in parenting assessment or intervention beyond a qualifying occupational therapy program			
	No	849	62.6
	Yes	344	25.4
	Unsure/can’t recall	164	12.1
Service type			
	Public (government) health service	682	50.3
	Independent private practice	425	31.3
	Private (for-profit) health service	371	27.3
	Non-government/charitable service	178	13.1
	Other	61	4.5
Primary reason for client referral/attendance at occupational therapy			
	Physical disability	872	64.3
	Mental health challenges	659	48.6
	Neurological disorders	651	48.0
	Intellectual disability	514	37.9
	Sensory disability	505	37.2
	Developmental disability	438	32.3
	No specific health condition or disability (e.g., social disadvantage)	160	11.8
	Other	92	6.8
Percentage of adult clients who are parents of children (0–18 years old)			
	0–20%	400	29.5
	21–40%	287	21.1
	41–60%	212	15.6
	61–80%	153	11.3
	81–100%	165	12.2
	I don’t know	134	9.9
	Missing data	6	0.4
Methods used to identify clients’ parental status			
I collect this data for every client as part of a routine assessment		875	64.5
I ask about parenting if a client has a condition/circumstance that might impact parenting		487	35.9
I find out about parenting if the client or another colleague brings it up		203	15.0
All clients in my service are parents		180	13.3
I would rarely be aware of a client’s parental status I use another method		96	7.1
		45	3.5

Most (*n* = 1,045, 77.0%) participants described themselves as female, had worked as an occupational therapist for 0–10 years (65.4%), and worked within a publicly (government) funded service context (50.3%). Most therapists worked with people with chronic disease or disability, with a small minority (*n* = 160, 11.8%) providing services to address occupational participation concerns unrelated to health or disability. Most participants (*n* = 1,013, 74.7%) had not undertaken or could not recall undertaking additional training or education relating to occupational therapy for parents.

### Do Occupational Therapists Ask About Parental Status?

As reported in Table 1, just under one-third of the sample (*n* = 400, 29.5%) indicated that between 0% and 20% of their adult clients were parents of children aged 0–18 years, while a smaller minority (*n* = 134, 9.9%) indicated they were unaware of what proportion of their clients were parents. The most frequently reported method of determining a client’s parental status was through routine collection of this data during the assessment process (*n* = 875, 64.5%), followed by questions posed to the client where the therapist perceived the client’s circumstances might impact upon parenting roles and occupations (*n* = 487, 35.9%). Again, a minority of respondents indicated they were rarely aware of a client’s parental status (*n* = 96, 7.1%).

### How Frequently Do Occupational Therapists Address Parenting as Part of Practice With Clients Who Are Parents?

Multinominal logistic regression models were developed to examine the relationship between (a) frequency of asking clients who were parents about parenting (see Table 2 for full details) and (b) frequency of working with clients who are parents on parenting roles and occupations (see Table 3 for full details) and the predictor variables region of practice; specific training in parenting assessment/intervention; years working as an occupational therapist; and percentage of adult clients who are parents.

**Table 2.** Multinomial Logistic Regression Results for Factors Predicting Frequency of Asking Clients About Parenting Roles.

Predictor	*B*	*SE*	Wald	df	*p*	Exp(*B*)	95% CI for Exp(*B*)
**Rarely or never (vs very frequently)**							
Intercept	−1.72	0.754	5.198	1	0.023	None	None
Training in parenting assessment/intervention (No)	0.924	0.456	4.116	1	0.042	2.52	[1.032, 6.155]
Region (Middle East and North Africa)	−0.296	0.558	0.282	1	0.596	0.743	[0.249, 2.221]
Region (East Asia and Pacific)	1.52	0.55	7.646	1	0.006	4.573	[1.557, 13.431]
Years of experience (0–2 years)	1.574	0.401	15.389	1	<0.001	4.824	[2.198, 10.589]
Proportion of clients who are parents (0–20%)	−1.03	0.333	9.569	1	0.002	0.357	[0.186, 0.686]
**Occasionally (vs. very frequently)**							
Intercept	−1.277	0.507	6.34	1	0.012	None	None
Training in parenting assessment/intervention (No)	0.214	0.253	0.713	1	0.398	1.239	[0.754, 2.035]
Region (Middle East and North Africa)	0.27	0.327	0.685	1	0.408	1.31	[0.691, 2.486]
Region (East Asia and Pacific)	1.007	0.348	8.354	1	0.004	2.737	[1.383, 5.417]
Years of experience (0–2 years)	1.334	0.317	17.706	1	<0.001	3.796	[2.039, 7.067]
Proportion of clients who are parents (0–20%)	−0.048	0.315	0.023	1	0.879	0.953	[0.514, 1.768]
**Frequently (vs. very frequently)**							
Intercept	−1.979	0.672	8.671	1	0.003	None	None
Training in parenting assessment/intervention (No)	0.392	0.326	1.451	1	0.228	1.48	[0.782, 2.803]
Region (Middle East and North Africa)	0.818	0.489	2.805	1	0.094	2.267	[0.870, 5.905]
Region (East Asia and Pacific)	1.477	0.499	8.75	1	0.003	4.38	[1.646, 11.657]
Years of experience (0–2 years)	0.541	0.385	1.975	1	0.16	1.717	[0.808, 3.650]
Proportion of clients who are parents (0–20%)	0.215	0.366	0.346	1	0.556	1.24	[0.605, 2.543]
**Sometimes (vs. very frequently)**							
Intercept	−0.759	0.473	2.574	1	0.109	None	None
Training in parenting Assessment/intervention (No)	0.106	0.239	0.198	1	0.657	1.112	[0.696, 1.778]
Region (Middle East and North Africa)	0.283	0.303	0.874	1	0.35	1.327	[0.733, 2.404]
Region (East Asia and Pacific)	0.928	0.321	8.363	1	0.004	2.529	[1.349, 4.744]
Years of experience (0–2 years)	0.419	0.281	2.226	1	0.136	1.52	[0.877, 2.634]
Proportion of clients who are parents (0–20%)	0.365	0.318	1.317	1	0.251	1.441	[0.772, 2.687]

**Table 3.** Multinomial Logistic Regression Results for Factors Predicting Frequency of Working With Parents on Parenting Roles or Occupations.

Predictor	*B*	*SE*	Wald χ²	df	*p*	Exp(*B*)	95% CI for Exp(*B*)
**Rarely or never (vs very frequently)**							
Intercept	−0.456	.516	0.78	1	.377	None	None
Training in parenting assessment/intervention (No)	1.141	0.299	14.53	1	<.001	3.13	[1.74, 5.63]
Region (East Asia and Pacific)	1.345	0.347	15.06	1	<.011	3.84	[1.95, 7.57]
Region (Europe and Central Asia)	1.23	0.381	10.42	1	.001	3.42	[1.62, 7.21]
Proportion of clients who are parents (41–60%)	−1.848	.340	29.51	1	<.001	0.16	[0.08, 0.31]
Proportion of clients who are parents (81–100%)	−2.475	0.372	44.27		<.001	0.08	[0.04, 0.17]
**Sometimes/occasionally (vs. very frequently)**							
Intercept	−0.392	0.434	0.82		.367	None	None
Training in parenting assessment/intervention (No)	0.488	0.206	5.62	1	.018	1.63	[1.09, 2.44]
Region (East Asia and Pacific)	1.009	0.312	10.48	1	.001	2.74	[1.49, 5.05]
Region (Europe and Central Asia)	1.096	0.340	10.40	1	.001	2.99	[1.54, 5.83]
Proportion of clients who are parents (81–100%)	–1.257	0.314	16.04	1	<.001	0.28	[0.15, 0.53]

When considering the frequency of asking clients who were parents about parenting, the model was statistically significant (χ^2^ = 273.05, df = 60, *p* < .001); however, the pseudo-*R*^2^ values (Nagelkerke *R*^2^ =.195) suggest a modest explanatory power. Likelihood ratio tests revealed that all four predictors significantly contributed to the model: training in parenting interventions, χ^2^(8) = 36.99, *p* < .001; region of practice, χ^2^(16) = 54.80, *p* < .001; years of experience as an OT, χ^2^(16) = 35.61, *p* = .003; and percentage of adult clients who are parents, χ^2^(20) = 119.37, *p* < .001.

Relative to occupational therapists who reported “very frequently” asking clients about parenting roles, those who reported doing so “rarely or never” were significantly more likely to work in the East Asia and Pacific region (odds ratio (OR) = 4.57, 95% confidence interval (CI) = [1.56, 13.43]), have 0–2 years of experience (OR = 4.82, 95% CI = [2.20, 10.59]), and to have a smaller proportion of clients who are parents (e.g., 21–40% of clients: OR = 0.06, 95% CI = [0.02, 0.15]).

Similarly, occupational therapists who reported “occasionally” asking about parenting were more likely to be in the East Asia and Pacific region (OR = 2.74, 95% CI = [1.38, 5.42]), have 0–2 years of experience (OR = 3.80, 95% CI = [2.04, 7.07]), and have a lower proportion of clients who are parents (e.g., 41–60%: OR = 0.40, 95% CI = [0.21, 0.78]).

Occupational therapists who reported “frequently” asking clients about parenting were more likely to be based in East Asia & the Pacific (OR = 4.38, 95% CI = [1.65, 11.66]) and less likely to report having received formal training in parenting (OR = 0.44, 95% CI = [0.21, 0.91]). Finally, therapists who selected “sometimes” were also more likely to be based in East Asia and Pacific (OR = 2.53, 95% CI = [1.35, 4.74]) and less likely to have received training (OR = 0.58, 95% CI = [0.35, 0.95]).

Due to the distribution of responses regarding the frequency of working with adult clients on parenting roles and occupations, the response categories of frequently and very frequently and “Sometimes/Occasionally” were combined, giving a dependent variable with three groups: “Rarely/Never,” “Sometimes/Occasionally,” and “Frequently/Very Frequently” (used as the reference category). The overall model was statistically significant, χ²(30) = 408.58, *p* < .001. Pseudo-R² values suggest moderate explanatory power (Nagelkerke R² = 0.298). Post-hoc likelihood ratio tests indicated that all predictor variables except years of experience contributed significantly to the model. Specifically, receipt of parenting-related training, χ²(4) = 86.45, *p* < .001, region, χ²(8) = 82.42, *p* < .001, and the proportion of clients seen who were parents χ²(10) = 114.96, *p* < .001) were each independently associated with frequency of engagement with parents, while years of experience was not statistically significant and thus was not retained in the interpretation of the model, χ²(8) =13.72, *p* = .089.

Relative to occupational therapists who reported “frequently/very frequently” working on parenting roles and occupations those who reported doing so “rarely/never” were significantly less likely to report formal training in parenting (OR = 3.13, 95% CI = [1.74, 5.63], *p* < .001), practice in East Asia and Pacific region (OR = 3.84, 95% CI = [1.95, 7.57], *p* < .001) or in Europe and Central Asia (OR = 3.42, 95% CI = [1.62, 7.21], *p* < .001). Furthermore, those occupational therapists with a higher proportion of adult clients who were parents were significantly less likely to fall in the “rarely/never” category; for example, for therapists with 41–60% of clients were parents (OR = 0.16, 95% CI = [0.08, 0.31], *p* < .001) and those reporting 81–60% of clients were parents (OR = 0.08, 95% CI = [0.04, 0.17], *p* < .001).

Compared with occupational therapists who “frequently/very frequently” worked on parenting roles and occupations, those participants who reported “sometimes/occasionally” doing so were more likely to lack specific parent-related occupational therapy training (OR = 1.63, 95% CI = [1.09, 2.44], *p* < .01) and practice in the East Asia and Pacific region (OR = 2.74, 95% CI = , *p* = .018) or in Europe and Central Asia (OR = 2.99, 95% CI = [1.54, 5.83], *p* = .001). For this comparison, the only significant caseload predictor was for therapists with 81–100% of clients who were parents. This group was less likely to fall into the “sometime/occasionally” category (OR= 0.28, 95% CI = [0.15, 0.53], *p* < .001).

### How Do Occupational Therapists Assess Parenting Performance?

Participants were asked to comment on the approach taken to the assessment of parenting performance. Most respondents (*n* = 661, 48.7%) reported rarely or never using formal assessment tools to evaluate the performance of parenting roles or occupations. Where formal assessments were conducted just over quarter of respondents (*n* = 366, 26.9%) described using generic occupational performance assessments such as the Canadian Occupational Performance Measure (COPM) (Law et al., 1990), while a smaller number of respondents reporting using standardized assessment tools designed either for their specific client population (*n* = 155, 11.4%) or for the general population of parents (*n* = 148, 10.9%).

### How Do Occupational Therapists Support Clients Who Are Parents in Relation to Parenting Roles and Occupations?

Respondents who reported working with clients on parenting concerns were asked to describe what aspects of parenting they provided support for, and how they delivered these interventions. The most frequently reported mode of intervention was via individual interventions tailored to the needs of clients (*n* = 809, 59.5%). This was followed by group programs for parents (*n* = 261, 19.2%) and structured individual parenting programs (*n* = 242, 17.8%). Table 4 provides an overview of the frequency with which participants described addressing specific parenting concerns and supporting parents in performing parenting tasks. Providing emotional support and/or problem-solving around parenting issues, teaching parenting tasks and skills, and referral of the parent to appropriate community supports for parenting were the most frequently used interventions, while prescription of adaptive equipment for parenting emerged as the least frequently used intervention.

**Table 4.** Frequency With Which Parenting Issues are Addressed and Support Provided to Parents to Complete Parenting Tasks (N = 1,196).

	Always		Usually		About half the time		Occasionally		Never	
Intervention	*n*	%	*n*	%	*N*	%	*n*	%	*n*	%
Prescribe adaptive equipment for parenting activities	163	12	243	17.9	142	10.4	235	17.3	213	15.7
Teach parenting tasks and skills	264	19.4	301	22.1	155	11.4	202	14.9	75	5.5
Support clients with parenting approaches and knowledge	241	17.7	280	20.6	162	11.9	191	14.0	122	9.0
Provide emotional support and/or problem-solving around parenting issues	332	24.4	311	22.9	168	12.4	156	11.5	29	2.1
Refer to or support the parent to engage with appropriate community supports for parenting	242	17.8	289	21.3	166	12.2	219	16.1	142	10.4
Advocate for their rights as parents	223	16.4	233	17.1	179	13.2	219	16.1	142	10.4
Work with other family members (e.g., children, partner) to support parenting	211	15.5	252	18.35	206	15.1	210	15.4	115	8.5
Other parenting support	131	9.6	162	11.9	111	8.2	92	6.8	491	36.1

## Discussion

Parenting is a highly complex and important occupational role (Lim et al., 2022). Occupational therapists can support parents to address difficulties in performing parenting occupations and recognize parenting as within the scope of professional practice (American Occupational Therapy Association, 2020; Canadian Association of Occupational Therapy, 2024). However, occupational therapy practice as it relates to parenting is poorly understood (McGrath et al., 2024). This study addresses this gap by providing, for the first time, a global understanding of occupational therapy assessment and interventions for adults relating to parenting roles and occupations. Our study, which provides data from occupational therapists working in 42 different countries, provides a first insight into how occupational therapists identify and respond to the parenting needs of their adult clients.

The lack of involvement in parenting by occupational therapists working with adults suggests practice remains heavily influenced by the medical model, which narrows therapists’ focus to individual impairments and personal functioning, excluding broader occupational roles like parenting (Turcotte & Holmes, 2023). This limited engagement may also reflect barriers identified by our participants (Honey et al., 2025), including unsupportive institutional structures, inadequate training and resources, limited assessment tools, and lack of recognition—among colleagues, parents, and therapists themselves—of occupational therapy’s potential to support parenting. Thus, despite frequently collecting data about parental status, most therapists do not routinely assess or intervene in parenting. This gap in practice is concerning, given the central role of parenting in adults’ lives and the significant number of parents who report difficulties or dissatisfaction with their performance of parenting occupations (Rusu et al., 2025). Such difficulties are associated with increased parental stress, which in turn is negatively associated with quality of life, life satisfaction, and happiness (Rusu et al., 2025). Furthermore, the impact of reduced capacity to engage in parenting occupations goes beyond the individual parent. Parenting practice impacts on any co-parents, the child(ren) being parented, as well as contributing to societal outcomes since parents are responsible for preparing children to contribute to communities in the future (McGrath et al., 2024). The failure of occupational therapists to attend to parenting when working with adult clients, therefore, represents a missed opportunity to support a critical aspect of individual, family, and community life.

Second, we sought to understand what approaches are used by occupational therapists to assess parenting roles and occupations. Our findings suggest that even among those occupational therapists who assess parenting, there is limited occupationally focused assessment practice and a narrow range of intervention approaches. The reliance on informal assessment methods and the absence of targeted occupational therapy assessment tools likely reflect the lack of a strong evidence base to support occupational therapy assessment with parents (Kirshbaum & Olkin, 2002; Lampe et al., 2019; Pastor-Bédard et al., 2023). While broad occupational performance measures can provide a useful starting point to assess parenting needs and concerns, there is an urgent need for occupational therapists to develop appropriate, occupational-focused means of evaluating parenting performance and satisfaction. Such measures are essential to clearly document parent needs and to inform interventions to support the performance of parenting roles. Given the significant influence of culture on parenting practices, care should be taken to ensure that these measures are sensitive to the sociocultural context in which parenting occurs and take into consideration parents’ own views on how parenting might be performed in the context of disability.

Finally, we wanted to consider what types of interventions are offered to parents to address concerns with parenting occupations and roles. Our results show that the most frequently reported interventions relate to the provision of emotional support and/or problem-solving around parenting issues. This is consistent with the reported expectations of people with physical disabilities who have been referred to occupational therapy (Honey et al., 2024). However, Honey et al. (2024) also reported that some people with physical disabilities found that occupational therapists lacked the professional knowledge and skills needed to address parenting goals. Similar concerns were identified by parents with neurological disorders (Pituch et al., 2024) and by occupational therapists themselves (Lampe et al., 2019). If occupational therapists are to provide effective practice, it is essential that this perceived knowledge gap is addressed. Furthermore, to ensure that intervention is informed by best available evidence, there is a need for research to establish what topics might be addressed by occupational therapists when providing emotional support and/or problem-solving around parenting issues and how the impact of these interventions should be evaluated.

Given that most of our participants reported working with adults with physical and sensory disabilities, it was surprising to see the relatively small proportion of participants who provided intervention in the form of provision of adaptive equipment to support the performance of parenting tasks. Previous research suggests that provision of assistive technology tailored to individuals’ needs, combined with modification of the home environment, can alleviate many of the difficulties associated with completing parenting tasks (Kaiser et al., 2012; National Council on Disability, 2012; Tuleja & DeMoss, 1999). However, research also reports that parents with disabilities encounter several barriers in accessing such equipment, including poor knowledge of potential options among health professionals, lack of availability of assistive technology, and limited funding to support purchase or hire of assistive devices to support parenting (Powell et al., 2019). To address these barriers, research is needed to support the development and testing of adaptive equipment to support parenting tasks across the lifespan. There is also a need for education to raise awareness among occupational therapists of the potential of adaptive equipment to support the performance of parenting tasks. Finally, occupational therapists have a role to play in advocating for the rights of people with disabilities to access adaptive equipment to support the performance of parenting activities and ultimately to ensure the rights of people with disabilities to parenthood are upheld.

Findings suggest that the frequency of working with parents to address parenting occupations and goals is associated with prior training, exposure to clients who are parents, and regional context. Early-career practitioners and those without additional training focusing on parents were more likely to report reduced engagement with parenting-related practice, highlighting the importance of targeted education in this domain. While the need for occupational therapy pre-professional curricula to include information about parenting roles and occupations has been previously identified (McGrath et al., 2024), a review of core textbooks for occupational therapy students indicates that parenting receives limited attention (Lim et al., 2022), suggesting there is a need for educational resources to be developed to support student learning. Such resources should address parenting at all stages (rather than focusing on early infant care) and should be careful to acknowledge the diversity of parenting practices rather than relying on existing understandings of parenting occupations, which are largely informed by research with White middle-class parents in Western contexts (Lim et al., 2022).

### Limitations

As with all studies, there are several limitations that should be considered when interpreting and applying the results. First, the use of convenience sampling means we are unable to calculate our survey response rate or determine the extent to which respondents are representative of occupational therapists working with adults. Participants in our survey are likely to have more interest in occupational therapy and parenting and a greater likelihood of addressing parenting in practice than other occupational therapists. Readers should also be reminded that our survey was developed for this study, and while we followed a comprehensive survey development process, it is possible that our questions were read and interpreted differently than how we intended. Furthermore, cultural variations in parenting beliefs, practices, and expectations may have influenced participants’ responses, potentially shaping how occupational therapists perceive and address parenting roles within their practice. Nevertheless, our study provides a first insight into global occupational therapy practice as it relates to parenting and highlights an urgent need to develop evidence-informed, parent-centric occupational therapy assessment and interventions.

## Conclusion

Parenting is recognized as a challenging, valued, and common adult role (Lim et al., 2022). While the potential for occupational therapists to support parenting is well recognized (McGrath et al., 2024), our study findings indicate a significant gap between what occupational therapists *could* offer parents who experience difficulty with parenting and what occupational therapists *do* offer.

## Supplemental Material

sj-docx-1-otj-10.1177_15394492251382465 – Supplemental material for Parenting and Occupational Therapy: An Exploration of Global Practice Supplemental material, sj-docx-1-otj-10.1177_15394492251382465 for Parenting and Occupational Therapy: An Exploration of Global Practice by Margaret McGrath, Anne Honey, Fidaa Almomani, Yu-Wei Ryan Chen, Yvonne Codd, Junghun Aj Kim, Masafumi Kunishige, Rodolfo Morrison, Jessica Peterson, Evelina Pituch, Muhammad Hibatullah Romli, Deena Rozen, Rachel Sabbah, Hassan I. Sarsak, Elaine Saunders, So Sin Sim, Hwei Lan Tan, Farahiyah Wan Yunus, Wong Wing Tung, Veronica O. Mara and John V. Rider in OTJR: Occupational Therapy Journal of Research
