# Supplementary material for: Parenting and Occupational Therapy: An Exploration of Global Practice
Source: OTJR (Thorofare N J). 2025 Nov 14;46(3):422–33. doi: 10.1177/15394492251382465 (PMC13219780; doi:10.1177/15394492251382465)
Supplement: sj-docx-1-otj-10.1177_15394492251382465 – Supplemental material for Parenting and Occupational Therapy: An Exploration of Global Practice [file sj-docx-1-otj-10.1177_15394492251382465.docx]

Appendix 1

**Supplemental Table 1**

*Participants’ Country of Practice (N=1357)*

| World Bank Region | Country | n | % |
| --- | --- | --- | --- |
| East Asia and Pacific |  |  |  |
|  | Australia | 130 | 9.6 |
|  | Japan | 57 | 4.2 |
|  | Malaysia | 36 | 2.7 |
|  | Singapore | 24 | 1.8 |
|  | New Zealand | 15 | 1.1 |
|  | Republic of Korea | 10 | 0.7 |
|  | Hong Kong | 9 | 0.7 |
|  | Taiwan | 2 | 0.1 |
|  | Philippines | 1 | 0.1 |
|  | Vietnam | 1 | 0.1 |
| Total |  | 285 | 21.0 |
| Middle East and North Africa |  |  |  |
|  | Jordan | 384 | 28.3 |
|  | Kingdom of Saudi Arabia | 99 | 7.3 |
|  | Israel | 25 | 1.8 |
|  | United Arab Emirates | 20 | 1.5 |
|  | Kuwait | 15 | 1.1 |
|  | Lebanon | 12 | 0.9 |
|  | Oman | 12 | 0.9 |
|  | Bahrain | 5 | 0.4 |
|  | Quatar | 7 | 0.5 |
|  | Palestine | 1 | 0.1 |
|  | Algeria | 1 | 0.1 |
| Total |  | 581 | 42.8 |
| Sub-Saharan Africa |  |  |  |
|  | South Africa | 3 | 0.2 |
|  | Kenya | 1 | 0.1 |
|  | Mauritius | 1 | 0.1 |
| Total |  | 5 | 0.4 |
|  |  |  |  |
| North America |  |  |  |
|  | Canada | 127 | 9.4 |
|  | United States | 76 | 5.6 |
| Total |  | 203 | 15.0 |
| South Asia |  |  |  |
|  | Pakistan | 2 | 0.1 |
|  | India | 1 | 0.1 |
| Total |  | 3 | 0.2 |
| Latin America and Caribbean |  |  |  |
|  | Chile | 83 | 6.1 |
|  | Argentina | 7 | 0.5 |
|  | Venezuela | 4 | 0.3 |
|  | Ecuador | 3 | 0.2 |
|  | Mexico | 1 | 0.1 |
|  | Dominican Republic | 1 | 0.1 |
| Total |  | 99 | 7.3 |
| Europe and Central Aisa |  |  |  |
|  | Republic of Ireland | 97 | 7.1 |
|  | United Kingdom | 64 | 4.7 |
|  | France | 4 | 0.3 |
|  | Austria | 3 | 0.2 |
|  | Finland | 1 | 0.1 |
|  | Greece | 1 | 0.1 |
|  | Portugal | 1 | 0.1 |
|  | Spain | 1 | 0.1 |
| Total |  | 172 | 12.7 |

Missing 9 0.7
